# A systematic review and meta-analysis of the prevalence of toxoplasmosis in hemodialysis patients in Iran

**DOI:** 10.4178/epih.e2018016

**Published:** 2018-04-23

**Authors:** Masoud Foroutan, Ali Rostami, Hamidreza Majidiani, Seyed Mohammad Riahi, Sasan Khazaei, Milad Badri, Elham Yousefi

**Affiliations:** 1 Department of Parasitology, Faculty of Medical Sciences, Tarbiat Modares University, Tehran, Iran; 2 Infectious Diseases and Tropical Medicine Research Center, Health Research Institute, Babol University of Medical Sciences, Babol, Iran; 3 Department of Epidemiology, School of Public Health, Shahid Beheshti University of Medical Sciences, Tehran, Iran; 4 Faculty of Health, Birjand University of Medical Sciences, Birjand, Iran; 5 Department of Medical Parasitology and Mycology, School of Public Health, Tehran University of Medical Sciences, Tehran, Iran

**Keywords:** *Toxoplasma gondii*, Seroprevalence, Hemodialysis patients, Iran

## Abstract

**OBJECTIVES:**

Toxoplasmosis is a parasitic disease that occurs worldwide, with a wide range of complications in immunocompromised patients. This systematic review and meta-analysis was performed to evaluate the seroprevalence of *Toxoplasma gondii* among patients undergoing hemodialysis in Iran.

**METHODS:**

We searched English and Persian databases for studies reporting *T. gondii* seroprevalence in Iranian hemodialysis patients through December 31, 2017. Inclusion and exclusion criteria were applied.

**RESULTS:**

A total of 10 studies containing 1,865 participants (1,048 patients and 817 controls) met the eligibility criteria. Immunoglobulin G (IgG) antibodies against *T. gondii* were found in 58% (95% confidence interval [CI], 46 to 70) of hemodialysis patients and 40% (95% CI, 31 to 50) of healthy controls, while immunoglobulin M (IgM) antibodies were found in 2% (95% CI, 0 to 6) of hemodialysis patients and 0% (95% CI, 0 to 1) of healthy controls. The meta-analysis showed that hemodialysis patients were significantly more likely to be seropositive for IgG (odds ratio [OR], 2.04; 95% CI, 1.54 to 2.70; p<0.001) and IgM (OR, 2.53; 95% CI, 1.23 to 5.22; p<0.001) antibodies against *T. gondii* infection than healthy individuals.

**CONCLUSIONS:**

The current study revealed a high prevalence of *T. gondii* infection in hemodialysis patients. Since hemodialysis patients are immunocompromised and *T. gondii* can cause serious clinical complications, we recommend that periodic screenings for *T. gondii* infection should be incorporated into the routine clinical care of these patients.

## INTRODUCTION

Toxoplasmosis is a cosmopolitan parasitic zoonosis caused by the well-known intracellular protist, *Toxoplasma gondii* [1,2]. Recently published systematic review articles have estimated the pooled prevalence of global *T. gondii* infection in various groups of humans. For instance, prevalence ranges of 0.8 to 77.5% in pregnant women and those of childbearing age [3] and 33.0% (95% confidence interval [CI], 28.0 to 39.0) among apparently healthy blood donors [4] were reported from a global perspective. Furthermore, in immunocompromised persons, such as HIV/AIDS patients, cancer patients, and transplant recipients, the estimated pooled prevalence of toxoplasmosis was reported to be 42.1% (95% CI, 34.0 to 50.2), 26.0% (95% CI, 20.5 to 31.5), and 42.1% (95% CI, 27.1 to 57.2), respectively [5].

Toxoplasmosis is frequently transmitted via congenital infections, ingesting oocyst-contaminated food or water, consuming raw or undercooked meat containing tissue cysts, and organ transplantation and blood transfusion through infected donors [6-9]. A wide range of ecological and behavioral risk factors, such as place of residence, geographical climate, and nutritional habits, have been implicated in parasite survival and dissemination [4,10-12]. Despite the asymptomatic and chronic nature of infection in immunocompetent individuals, toxoplasmosis may result in life-threatening outcomes in at-risk people such as pregnant women and immunocompromised individuals, including people undergoing radiation therapy, cancer patients, HIV-positive individuals, transplant recipients, multi-transfused thalassemia patients, and hemodialysis patients [13-19]. Having potential neurotropism, the main effects of the parasite are brain damage, neurological defects, and even encephalitis in immunodeficient people [13,20-24]. Unfortunately, no commercially licensed vaccine is available to prevent toxoplasmosis in humans [25,26].

The kidneys play a central role in maintaining body homeostasis via blood filtration. Without excretion, waste substrates and toxins would accumulate in the body and endanger the individual’s life. Therefore, kidney transplant or hemodialysis is indispensable in individuals with renal failure [27]. According to reports, the number of people with renal failure and end-stage renal disease requiring hemodialysis has increased during the last 2 decades [28]. Patients undergoing hemodialysis are considered to be immunocompromised, particularly due to immune response dysfunctions regarding phagocytosis, chemotaxis, and the complement system [29]. Hence, these individuals are more vulnerable to opportunistic pathogens such as *T. gondii* [30].

Several articles have investigated the prevalence of *T. gondii* infection in hemodialysis patients in Iran. Herein, we present a systematic review and meta-analysis that was designed to determine the exact prevalence of the infection among this population.

## MATERIALS AND METHODS

### Study area

Covering a wide area in the Middle East (1,648,195 km^2^ ), Iran has a population of approximately 80 million (as of 2015), and is located between 25°3ʹ and 39°47ʹN and 44°5ʹ and 63°18ʹE, bordering Iraq and Turkey to the west, Afghanistan and Pakistan to the east, the Persian Gulf and Oman Sea to the south, and Azerbaijan, Armenia, and Turkmenistan to the north. Except for a small region on the margin of the Caspian Sea coast with considerable annual rainfall that is covered by dense vegetation, the general climate of Iran is hot and dry, forming the Iranian plateau. It is one of the world’s most mountainous countries, and its landscape is dominated by rugged mountain ranges that separate various basins and plateaus from each other. The populous western part is the most mountainous, with ranges such as the Caucasus, Zagros, and Alborz Mountains. Lower temperatures, severe winters, and heavy snowfall occur in the Zagros basin, while in the central and eastern basins there is an arid climate because of high-altitude mountain ranges in the western and northern parts. These mountain ranges are so high that rain clouds cannot reach the central and eastern basins. Annual precipitation is 680 mm in the eastern part of the plain and more than 1,700 mm in the western part [31].

### Search strategy

**Figure 1.**
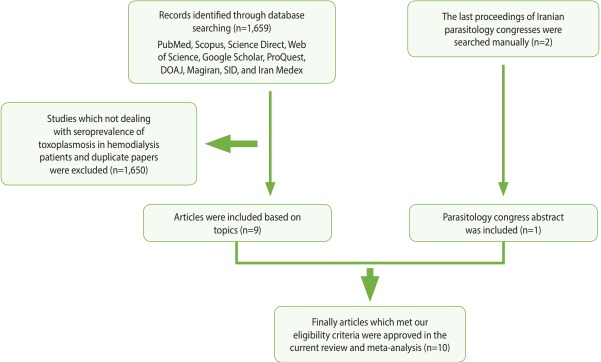
Flowchart describing the study design process. DOAJ, Directory of Open Access Journals; SID, Scientific Information Database.

In order to assess the prevalence of *Toxoplasma* infection among hemodialysis patients in Iran, compared with healthy persons, we searched for relevant papers in 7 English-language databases (PubMed, Scopus, Science Direct, Web of Science, Google Scholar, ProQuest, and the Directory of Open Access Journals) and three Persian-language databases (Magiran, Scientific Information Database, and Iran Medex) from their inception until December 31, 2017. Additionally, the proceedings of the last 2 Iranian parasitology congresses were browsed manually (Figure 1). This systematic review was conducted using Medical Subject Heading terms, including: “*Toxoplasma*,” “toxoplasmosis,” “*Toxoplasma gondii*,” “hemodialysis patients,” “renal failure,” “dialysis,” “epidemiology,” “prevalence,” and “Iran,” alone or in combination with “OR” and/ or “AND” operators.

### Study selection and data extraction

The initial citations obtained during database exploration were recorded in a Word file according to their topics and abstracts. Following primary screening, potentially eligible records were selected for full-text download. The final eligibility and inclusion criteria for the downloaded full texts were appraised by 2 independent reviewers (MF and SK). The selected articles were scrutinized and discrepancies between the reviewers were addressed by discussion and consensus with a third reviewer (MB). Subsequently, an author (MF) extracted the requisite data, and the others (SK and MB) rechecked them. The following inclusion criteria were applied in the current systematic review: (1) original research papers, short reports, or letters to the editor; (2) case-control studies that estimated the prevalence of toxoplasmosis in hemodialysis patients and healthy individuals; (3) studies published in English or Persian; (4) studies published online before December 31, 2017; (5) studies for which the full texts were available (abstracts from the last 2 Iranian parasitology congresses were also acceptable); and (6) studies with information on the exact total sample size and positive samples in the case and control groups. Each paper that did not meet the above-mentioned criteria was excluded. Additionally, the references of the selected papers were hand-checked to find relevant articles that were not retrieved in the database search. Ultimately, the following characteristics of each relevant study were extracted: first author, province, year of publication, sample size, seroprevalence of anti-*Toxoplasma* immunoglobulin G (IgG) and/or immunoglobulin M (IgM) antibodies in the case and control groups, the overall prevalence, matching (age, gender, or both), and age range or mean age. Moreover, details such as the diagnostic method, the cutoff value or antibody titer for serologic methods, the name of the kit that was used, results, and/or suggestions also were extracted. The Preferred Reporting Items for Systematic Reviews and Meta-Analyses criteria were employed to report our findings [32].

### Study quality assessment

The Newcastle-Ottawa Scale was employed to assess the quality of the included papers [33]. A score with a maximum of 9 points was given for 8 items in 3 different categories, including the subject selection criteria (0-4 points), the comparability of subjects (0-2 points), and exposure (0-3 points). Briefly, a study could be awarded a maximum of 1 point for each numbered item within the selection and exposure categories. Additionally, a maximum of 2 points could be given for comparability. Papers with a total score of 0-3, 4-6, and 7-9 points were considered to be of poor, moderate, and high quality, respectively [34].

### Meta-analysis

The meta-analysis procedure was done as previously described [34-39]. Briefly, for each included study, the common odds ratio (OR) and respective 95% CIs were estimated. The results of the meta-analysis were visualized as a forest plot representing the prevalence estimates and related CIs of each study, along with summary measures. Heterogeneity was also analyzed using the Cochran Q and I^2^ statistics. Furthermore, a funnel plot based on the Egger regression test was used to explore publication bias and small study effects.

## RESULTS

**Figure 2.**
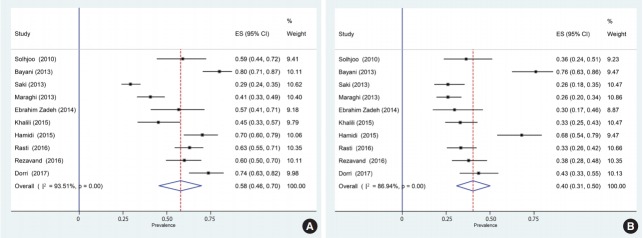
Forest plot diagram of the present systematic review and meta-analysis based on immunoglobulin G antibodies in case (A) and control (B) groups. ES, effect size; CI, confidence interval.

**Figure 3.**
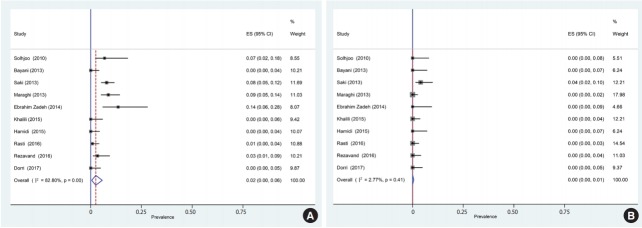
Forest plot diagram of the present systematic review and meta-analysis based on immunoglobulin M antibodies in case (A) and control (B) groups. ES, effect size; CI, confidence interval.

Of the 1,659 identified articles, 10 were included, and the results from these studies were weighted (Table 1) [40-49]. One study was removed due to over-matching [50]. A flow diagram showing the study selection process is presented in Figure 1. All the included studies had a case-control design, and the number of the cases and controls was 1,048 and 817, respectively, with a total of 1,865 participants across the 10 studies included. The main characteristics and results of the included studies are shown in Table 1. IgG *T. gondii* antibodies were reported in 58% (95% CI, 46 to 70) of the hemodialysis patients and in 40% (95% CI, 31 to 50) of the healthy controls (Figure 2), while IgM antibodies were found in 2% (95% CI, 0 to 6) of the hemodialysis patients and 0% (95% CI, 0 to 1) of the healthy controls (Figure 3). Detailed information about the studies, including the diagnostic method, cutoff value or antibody titer, and name of the kit, is presented in Supplementary Material 1.

**Figure 4.**
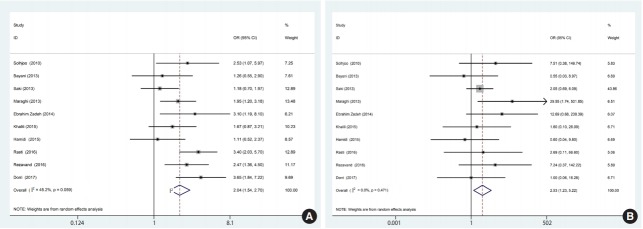
Forest plot of ORs related to the case (A) and control (B) groups. OR, odds ratio; CI, confidence interval.

**Figure 5.**
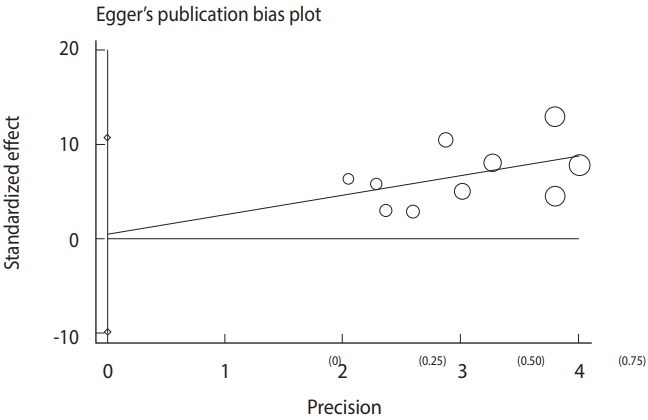
Egger plot for detecting publication bias. The parentheses indicate the inverse of sample size.

Based on the results of the meta-analysis, patients undergoing hemodialysis were significantly more likely to be seropositive for IgG (OR, 2.04; 95% CI, 1.54 to 2.70; p<0.001) and IgM (OR, 2.53; 95% CI, 1.23 to 5.22; p< 0.001) antibodies against *T. gondii* infection than healthy individuals (Figure 4). The heterogeneity among the studies for IgG (χ^2^ = 16.42; I^2^ = 45.2%; 95% CI, 0 to 74) and IgM (χ^2^ = 8.64; I^2^ = 0.0%; 95% CI, 0 to 62) antibodies was acceptable. To identify publication bias, we used Egger plots (Figure 5). According to the symmetry assumption, no significant publication bias was found in studies presenting results for the IgG (p= 0.92) and IgM (p= 0.33) antibodies.

## DISCUSSION

Elevated levels of blood urea in patients suffering from chronic kidney disease could lead to dysfunction of immunological factors such as polymorphonuclear leukocytes, nitric oxide, and platelets [30], thereby weakening the immune system and increasing the risk of infection, which is the cause of 40% of deaths in these patients [51]. Dialysis is essential to remove waste materials, excess water, and urea from the blood, and therefore plays a vital role in promoting survival and improving the quality of life of these patients [29]. Therefore, screening for opportunistic infections such as toxoplasmosis, identifying the potential risk factors for such infections, and ensuring early treatment in such patients would be useful.

The present meta-analysis assessed the seroprevalence of *T. gondii* infection in hemodialysis patients in Iran. The results demonstrated a relatively high prevalence (58%) of *T. gondii* infection in hemodialysis patients, which was significantly higher than was observed in healthy controls. The prevalence reported here generally agrees with other studies of Iranian immunocompromised individuals, including transplant recipients (55%) and HIV/AIDS patients (50%) [52]. However, this rate is significantly higher than the mean (39%) seroprevalence of *T. gondii* infection previously reported in the general population and cancer patients in Iran (45%) [11,52]. Moreover, the prevalence we observed in hemodialysis patients is in accordance with the reports of Yazar et al. [53] from Turkey (56.0%), which borders Iran, Aufy et al. [54] from Egypt (56.0%), and Alvarado-Esquivel et al. [55] from Mexico (56.7%).

Regarding acute infections, our results indicated that 2.0% of Iranian patients undergoing hemodialysis were seropositive for IgM. This result is consistent with the rate reported by Yazar et al. [53] in Turkey (1.7%), but lower than that reported by Aufy et al. [54] in Egypt (16.7%). Moreover, among the included studies, only 3 [42,47,48] applied molecular methods (polymerase chain reaction) to detect *T. gondii* infection. In all those studies, active infection (circulating DNA) was only observed in hemodialysis patients, whereas no controls showed positive results [42,47,48]. This result indicates that hemodialysis patients were more susceptible to acquire *T. gondii* infection. This trend could have been due to the inability of these patients to ensure appropriate personal and food hygiene. Considering the immune system dysfunction in these patients, acute or active infection could result to life-threatening complications, such as encephalitis.

The comprehensive literature search, rigorous methodology, duplicated data extraction and quality assessment by 2 independent reviewers, clear inclusion and exclusion criteria, and the absence of publication bias are strengths of this meta-analysis. Nonetheless, there are some limitations of our study that are due to the nature of the studies that were included. In case-control studies, selecting the controls is an important pitfall. Some of the included studies did not apply matching for case and controls, and in the majority of the included studies, the healthy controls were drawn from an undefined setting. Moreover, the majority of the studies included in this meta-analysis did not provide data about the duration of dialysis; therefore, we were unable to assess the effect of this important factor on the prevalence of infection. Another issue could be variation in the sensitivity and specificity of enzyme-linked immunosorbent assay kits and the different cutoff values that were used to detect IgG and IgM antibodies. Furthermore, most of the included studies did not evaluate risk factors, which is an important issue regarding the acquisition of *T. gondii* infection in immunocompromised patients. It has been well demonstrated that suppression of the host immune response is an important risk factor for the reactivation of chronic infections. Other important risk factors for *T. gondii* infection are intake of sporulated oocysts from contaminated water or unwashed fruit and vegetables, keeping cats indoors as pets, and the consumption of raw or undercooked meat containing *T. gondii* tissue cysts [1,4,10,12]. The geographical area is also an important factor associated with the prevalence of *T. gondii* infection [12]. In Iran, the highest prevalence of *T. gondii* infection was observed in northern areas. In the present meta-analysis, the study by Bayani et al. [41] that was performed in Mazandaran Province (north Iran) reported the highest prevalence of *T. gondii* infection in cases and controls.

### Recommendations

Some recommendations for the better management and treatment of hemodialysis patients and subsequent studies are given below: (1) We suggest that periodic screenings for *T. gondii* infection should be incorporated into the routine clinical care of hemodialysis patients; (2) Measures to prevent the acquisition of *T. gondii* infection, such as eating well-cooked meat and well-cleaned vegetables, are also recommended; (3) More studies are needed to further understand the prevalence of *T. gondii* infection and the impact thereof on the health of hemodialysis patients in regions where studies of this subject have not been carried out; and (4) We recommended that a standard questionnaire be designed for a more comprehensive assessment of related risk factors, including place of residence, gender, the duration of hemodialysis, education level, blood group, occupation, and history of immune suppression.

## CONCLUSION

The results of this study revealed that acute and chronic *T. gondii* infections were more frequent in hemodialysis patients than in healthy controls. Since patients undergoing hemodialysis are immunocompromised, *T. gondii* infections, especially in the acute phase, could cause serious clinical complications.

## Figures and Tables

**Table 1.** Characteristics of the included studies

Study	Province	Year	Group	Sample size	Seroprevalence		Age (range or mean±SD)	Matching	Quality score
					IgG	IgM			
Solhjoo et al. [40]	Fars	2010	Case	44	26 (59.1)	3 (6.8)	59.1-14.3	Age and gender	7
			Control	44	16 (36.4)	0 (0.0)	59.0-14.3		
Bayani et al. [41]	Mazandaran	2013	Case	90	72 (80.0)	0 (0.0)	42.5-11.5	ND	5
			Control	50	38 (76.0)	0 (0.0)	34.8-10.4		
Saki et al. [42]	Khuzestan	2013	Case	280	82 (29.3)	22 (7.9)	16.0-80.0	Gender	6
			Control	100	26 (26.0)	4 (4.0)	-		
Maraghi et al. [43]	Khuzestan	2013	Case	150	61 (40.7)	13 (8.7)	21.0-87.0	ND	5
			Control	150	39 (26.0)	0 (0.0)	-		
Ebrahim Zadeh et al. [44]	Sistan and Baluchistan	2014	Case	37	21 (56.7)	5 (13.5)	17.5±4.1	Age and gender	7
			Control	37	11 (29.7)	0 (0.0)	20.0±5.2		
Khalili et al. [45]	Chaharmahal and Bakhtiari	2015	Case	62	28 (45.0)	0 (0.0)	-	Age	4
			Control	100	33 (33.0)	0 (0.0)	-		
Hamidi et al. [46]	East Azerbaijan	2015	Case	84	59 (70.2)	0 (0.0)	54.3±12.6	Age and gender	6
			Control	50	34 (68.0)	0 (0.0)	55.5±8.2		
Rasti et al. [47]	Isfahan and Qom	2016	Case	135	85 (63.0)	1 (0.7)	58.6±16.1	Age	4
			Control	120	40 (33.3)	0 (0.0)	52.8±17.0		
Rezavand et al. [48]	Tehran	2016	Case	90	54 (60.0)	3 (3.3)	43.8±17.1	Age and gender	5
			Control	90	34 (37.8)	0 (0.0)	41.8±18.9		
Dorri et al. [49]	Sistan and Baluchistan	2017	Case	76	56 (73.7)	0 (0.0)	-	ND	4
			Control	76	33 (43.4)	0 (0.0)	-		

Values are presented as number or number (%). IgG, immunoglobulin G; IgM, immunoglobulin M; SD, standard deviation; ND, not described.

## SUPPLYMENTARY MATERIALS

Supplementary Material 1: Table S1 is available at http://www.e-epih.org/.
