# Supplementary material for: A systematic review and meta-analysis of the prevalence of toxoplasmosis in hemodialysis patients in Iran
Source: Epidemiol Health. 2018 Apr 23;40:e2018016. doi: 10.4178/epih.e2018016 (PMC6060338; doi:10.4178/epih.e2018016)
Supplement: Supplementary file 1 [file epih-40-e2018016-supplementary.pdf]

Table S1. Diagnostic methods

Study	Province	Method	Cutoff value (antibody titer)	Kit/manual
(Solhjoo *et al.*, 2010) [A01]	Fars	ELISA	-	DIA.PRO (Diagnostic Bioprobes Srl. [20128]-Italy)
(Bayani *et al.*, 2013) [A02]	Mazandaran	ELISA	-	Trinity Biotech Captia, USA
(Saki *et al.*, 2013) [A03]	Khuzestan	ELISA	The IgG and IgM levels equal or higher than 1.1 IU/mL were reported as a positive.	Torch-IgG, IgM-Trinity Biotech Company, USA
(Maraghi *et al.*, 2013) [A04]	Khuzestan	ELISA	IgG: 1 – 4: positive, > 4: high positive<br>IgM: ≥ 1.11: positive	Orgenium Laboratories-Finland
(Ebrahim Zadeh *et al.*, 2014) [A05]	Sistan and Baluchistan	ELISA	IgG: Serum level more than 10 IU/mL was considered positive.<br>IgM: serum level of more than 1 IU/mL was considered positive.	Kit (ADAlist Italia)
(Khalili *et al.*, 2015) [A06]	Chaharmahal and Bakhtiari	ELISA	-	Kit (A-prodiagnostic)
(Hamidi *et al.*, 2015) [A07]	East Azerbaijan	ELISA	-	Acon ELISA kit
(Rasti *et al.*, 2016) [A08]	Isfahan and Qom	ELISA	> 11 IU/mL	Pishtaz Teb, Tehran, Iran
(Rezavand *et al.*, 2016) [A09]	Tehran	ELISA		VIROIMMUN, Germany
(Dorri *et al.*, 2017) [A10]	Sistan and Baluchistan	ELISA	-	Pars Azmoon, Iran

REFERENCES

A01. Solhjoo K, Jahromi AS, Parnian-Rad A. Anti-Toxoplasma gondii antibodies in haemodialysis patients. Am J Infect Dis 2010;6:13-17.

A02. Bayani M, Mostafazadeh A, Oliaee F, Kalantari N. The prevalence of Toxoplasma gondii in hemodialysis patients. Iran Red Crescent Med J 2013;15:e5225.

A03. Saki J, Khademvatan S, Soltani S, Shahbazian H. Detection of toxoplasmosis in patients with end-stage renal disease by enzyme-linked immunosorbent assay and polymerase chain reaction methods. Parasitol Res 2013;112:163-168.

A04. Maraghi S, Yadyad MJ, Sheikhi M, Shamakhteh F, Latifi SM. Study the anti-Toxoplasma antibodies (IgG and IgM) in hemodialysis patients of Abadan and Khoramshahr cities Southwest Iran in 2011 using ELISA. Jundishapur J Microbiol 2013;6:e7113.

A05. Ebrahim Zadeh A, Bamedi T, Etemadi S, Shahrakipour M, Saryazdipour K. Toxoplasmosis as a complication of transfusion in hemodialysis patients. Iran J Ped Hematol Oncol 2014;4:22-25.

A06. Khalili B, Mortezaei S, Fazeli M. The comparison of anti-Toxoplasma antibody (IGM, IgG) In hemodialysed patients and those undergoing chemoterapy with healthy blood donor, shahr-E-kord, 1392. Iran J Parasitol 2015;10(Suppl 1):311.

A07. Hamidi F, Etemadi J, Mehrabani NG, Oskouei MM, Motavalli R, Ardalan MR. Comparison of Toxoplasma gondii seropositivity in hemodialysis and peritoneal dialysis patients. J Coast Life Med 2015;3:621-622.

A08. Rasti S, Hassanzadeh M, Soliemani A, Hooshyar H, Mousavi SG, Nikoueinejad H, et al. Serological and molecular survey of toxoplasmosis in renal transplant recipients and hemodialysis patients in Kashan and Qom regions, central Iran. Ren Fail 2016;38:970-973.

A09. Rezavand B, Poornaki AM, Mokhtari KR, Mohammad A, Andalibian A, Abdi J. Identification and determination of the prevalence of Toxoplasma gondii in patients with chronic renal failure by ELISA and PCR. Asian Pac J Trop Dis 2016;6:347-349.

A10. Dorri M, Dabirzadeh M, Maroufi Y, Afshari M, Chokamy MB. Prevalence of anti-Toxoplasma IgG and IgM in hemodialysis patients comparing to healthy individuals in Sistan area, Iran. J Nephropharmacol 2017;6:106-109.
